# Resection of an Orbital Apex Cavernous Hemangioma Adhering to the Oculomotor Nerve via Five-Hand Endonasal and Transcaruncular Approaches

**DOI:** 10.7759/cureus.85146

**Published:** 2025-05-31

**Authors:** Lorenzo Ripetta, Kinga Yo, Tessei Kuruma, Chrisha Faye T Habaluyas, Yasuhiro Takahashi

**Affiliations:** 1 Oculoplastic, Orbital and Lacrimal Surgery, Aichi Medical University Hospital, Nagakute, JPN; 2 Otorhinolaryngology, Aichi Medical University, Nagakute, JPN

**Keywords:** endonasal approach, five-hand technique, oculomotor nerve, orbital apex tumor, transcaruncular approach

## Abstract

A 43-year-old male presented with marked loss of visual acuity in the left eye during a two-year follow-up for a cavernous hemangioma located in the inferomedial orbital apex. A four-handed endonasal and transcaruncular orbital tumor resection was planned and carried out under general anesthesia. During the surgery, the oculomotor nerve branch was found to be firmly adherent to the tumor. To facilitate safe dissection, an additional surgeon provided a fifth hand by inserting a cotton swab and seeker through the nose, enabling precise separation of the oculomotor nerve branch from the tumor. A complete excision of the tumor was achieved, leading to full recovery of visual acuity and no paralysis of the medial rectus muscle at the six-month follow-up. This case highlights the effectiveness of a five-handed endonasal and transcaruncular approach for resection of orbital apex tumors.

## Introduction

The excision of tumors in the inferomedial orbital apex region can be tough to approach because of the delicate structures that course through it [1]. Surgeons prioritize avoiding damage to the optic nerve, as well as the oculomotor nerve, trochlear nerve, extraocular muscles, and ophthalmic artery, to preserve the patient’s vision and ocular motility [1]. The endonasal approach provides a direct route to the medial aspect of the orbital apex without crossing the path of the optic nerve [1-3]. Although the two-hand technique of handling an endoscope with one hand and one instrument with the other hand is commonly applied for excision of such tumors, four-handed techniques via the nostrils on both sides or combined endonasal and transcaruncular approaches have been reported for more secure dissection of tumors from the surrounding orbital soft tissue [3-6].

Nevertheless, since the oculomotor nerve branch runs onto the inside surface of the medial rectus muscle [7], tumor dissection is occasionally difficult when this nerve firmly attaches to a tumor located in the inferomedial orbital apex. Forceful dissection between a tumor and the oculomotor nerve can cause contusion and laceration of the nerve, potentially leading to persistent diplopia. Herein, we report a case of an orbital hemangioma located in the inferomedial orbital apex that was strongly adherent to the oculomotor nerve branch. To achieve a safe and complete resection, we employed a novel five-hand endonasal and transcaruncular approach, which allowed precise nerve detachment from the tumor and minimized the risk of postoperative complications.

## Case presentation

A 43-year-old man consulted with our institution to treat left ptosis. The patient underwent a craniotomy to approach an orbital apex tumor in the left inferomedial orbit by a neurosurgeon at another clinic two months before referral to our institution. However, the tumor was only partially removed, even though the superior rectus/levator palpebrae superioris muscles were incised to secure the surgical field. The pathological diagnosis was cavernous hemangioma. The past medical history and family history were unremarkable.

At the initial consultation, the best-corrected visual acuity was 1.2 in both eyes, and the intraocular pressure was 12 mm Hg and 10 mm Hg in the right and left eyes, respectively. Margin reflex distance-1 (MRD-1) was 3.5 mm on the right side and -2 mm on the left side, and left levator function was 5 mm. Supraduction in the left eye was moderately restricted. Hertel exophthalmometric value was 15 mm in both eyes (base, 102 mm).

Magnetic resonance imaging (MRI) showed a tumor surrounded by the optic nerve and medial and inferior rectus muscles on the left side. The width of the superior rectus/levator palpebrae superioris muscles on the left side was 2 mm shorter compared to that on the right side.

The patient refused surgical resection of the tumor because of being busy with work. However, the patient noticed vision loss in the left eye two years after the initial consultation.

**Figure 1:**
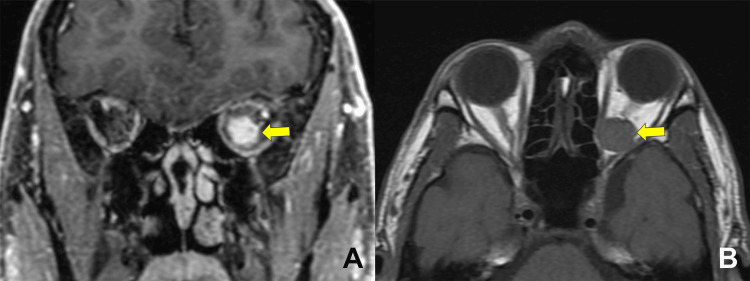
Preoperative magnetic resonance images. A T1-weighted enhanced coronal (A) and axial images (B) showing an orbital apex tumor (arrows) surrounded by the optic nerve and medial and inferior rectus muscles.

The best-corrected visual acuity was 10 cm/hand motion in the left eye, and the relative afferent pupillary defect was positive in the left eye. Funduscopic examination revealed a mildly pale disc in the left eye. MRD-1, extraocular muscle motility, and Hertel exophthalmometric values were unchanged. MRI revealed enlargement of the orbital tumor, which compressed the optic nerve (Figure 1). 

**Figure 2:**
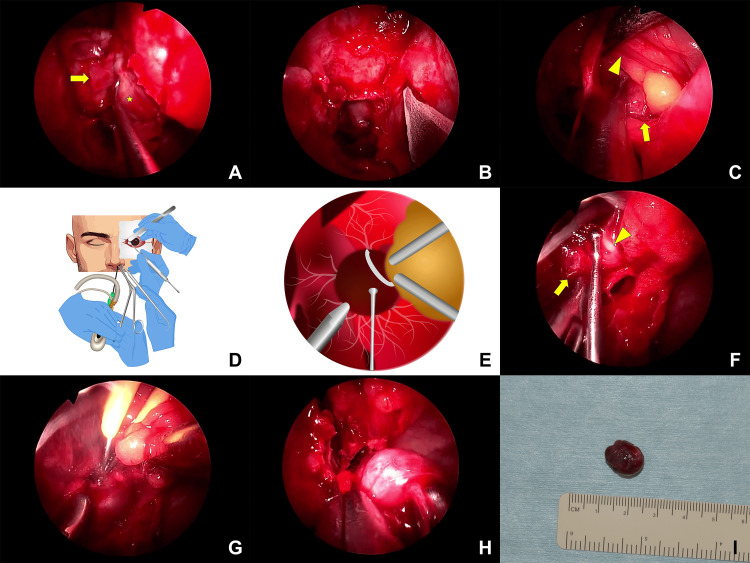
Intraoperative photographs and schemata. A: the medial orbital wall (arrow) was removed; B: the periosteum was incised; C: the medial rectus muscle (arrowhead) was retracted to expose the tumor (arrow); D: a schema of the five-hand approach; E, F: a hand with a seeker was added to dissect the nerve (arrowhead) and tumor (arrow); G: small vessels flowing into the tumor were coagulated; H: the tumor was extracted from the nostril; I: the excised tumor. Figures D and E are created by the authors using Editage (Cactus Communications, NJ, USA)

**Figure 3:**
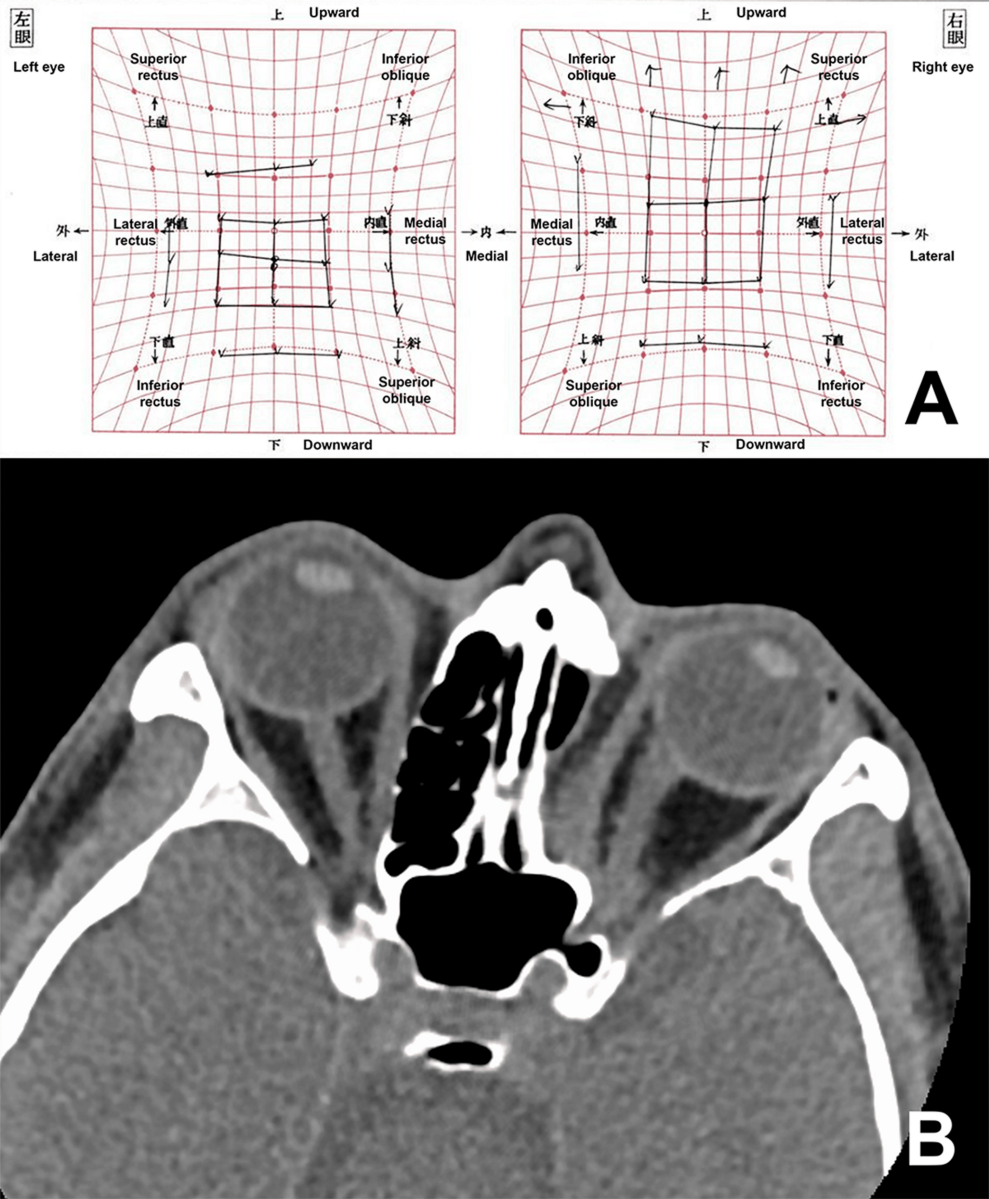
Postoperative findings. A: Hess chart showing no restriction of adduction; B: an axial computed tomographic image taken nine months after surgery showing no recurrence of the tumor and no reconstruction of the medial orbital wall.

First, the four-handed endoscopic endonasal and transcaruncular approaches were started for the resection of the orbital apex tumor under general anesthesia by one ENT surgeon (KY) and one oculoplastic surgeon (YT). Radical surgery was performed in the ethmoidal and sphenoidal sinuses. The frontal and maxillary sinuses were opened to avoid secondary sinusitis caused by prolapsed orbital soft tissues. The medial orbital wall (lamina papyracea) was removed (Figure 2A). After an incision of the periosteum (Figure 2B), the transcaruncular approach was performed. The lacrimal caruncle was incised using Westcott tenotomy scissors. Stevens' tenotomy scissors were used for blunt dissection towards the part 5 mm posterior to the posterior lacrimal crest. The medial rectus muscle was retracted using forceps, and a malleable retractor was inserted from the wound in the lacrimal caruncle to expose the orbital tumor (Figure 2C). During tumor extraction, the medial rectus muscle branch of the oculomotor nerve was found to adhere to the tumor. The hand of another ENT surgeon (TK) with a seeker or cotton swab inserted into the nostril was added for dissection between the nerve and tumor (Figures 2D-2F). The hand manipulated a bipolar coagulator to coagulate small vessels flowing into the tumor (Figure 2G). The tumor was pulled out via the nostril (Figures 2H, 2I). The medial orbital wall was not reconstructed for optic nerve decompression (Figure 3) [5]. The conjunctiva and caruncle were closed with interrupted 6-0 polyglycolic acid sutures (Vsorb, Kono Seisakusho Co., Ltd., Tokyo, Japan). Finally, the nasal wound was packed with an alignment wound dressing. Pathological findings of the excised tumor corresponded to cavernous hemangioma.

Three days after surgery, the visual acuity improved to 0.8 in the left eye, but it did not move in the medial direction due to oculomotor nerve paralysis, for which three-cycle steroid pulse therapy was performed.

At the nine-month follow-up, the visual acuity was 1.2 in the left eye. Although supraduction was still restricted, adduction was normal in the left eye (Figure 3A). MRD-1 was 4 mm on the right side and 0.5 mm on the left side. The tumor did not recur (Figure 3B).

## Discussion

To our knowledge, this is the first reported case in which we applied a five-hand technique for resection of a cavernous hemangioma located in the inferomedial orbital apex. Orbital cavernous hemangiomas can be removed by simple extraction using forceps and a cryoprobe [8]. Therefore, we started to remove an apical cavernous hemangioma by using one hand via the nostril to manipulate the endoscope, another hand via the nostril to hold the tumor with forceps, and two hands via a transcaruncular approach using forceps and a retractor to mobilize the medial rectus muscle and orbital fat to expose the tumor surface in the superior and inferior directions. However, given the strong adhesion of the tumor to the oculomotor nerve branch, the conventional four-hand approach was insufficient for safe and controlled dissection. An additional fifth hand by another ENT surgeon inserted a cotton swab and seeker through the nose, allowing delicate separation of the oculomotor nerve branch from the tumor. This fifth hand also manipulated a bipolar coagulator to coagulate the small vessels supplying the tumor.

The introduction of the five-hand technique proved instrumental in achieving a safe and complete tumor resection while minimizing iatrogenic nerve damage. The counteraction applied to the tumor facilitated controlled dissection, ensuring the oculomotor nerve was preserved without excessive traction or injury. Postoperatively, the patient’s vision fully recovered, and although transient oculomotor nerve palsy was observed, visual function improved with steroid therapy, and the tumor did not recur during the follow-up period.

A binarial bimanual approach is an alternative, useful technique for orbital apical tumor resection located in the medial orbit. Soft tissues adhering to an orbital tumor can be detached using instruments inserted from the nostril on the unaffected side. This surgery can be performed by ENT surgeons only [5]. However, this surgery is more invasive because radical surgery in the ethmoidal and sphenoidal sinuses and removal of the intersinus septum of the sphenoid sinus on the unaffected side are necessary to connect the affected and unaffected sinuses [9].

The five-hand technique may have a limited indication for cases of an orbital tumor strongly adhering to the surrounding tissue. A four-hand technique is sufficient for the removal of an apical tumor without adhesion of surrounding tissues. In addition, the five-hand approaches are, in general, performed by a multidisciplinary team of ENT and oculoplastic surgeons, which is feasible only in limited institutions [5].

## Conclusions

Despite cavernous hemangiomas being the most common primary tumors of the orbit, mass lesions of the orbit as a whole are rare, which is why we believe that this report serves the purpose of not only adding to the collective database regarding these entities but also providing an example of a modification of the already described four-hand approach. The five-hand technique may prove valuable for challenging cases, particularly in instances of strong tumor-nerve adherence. Further studies and case series may help establish its broader applicability in orbital surgery.
